# Case report: Medical student types journals during ketamine infusions for suicidal ideation, treatment-resistant depression, post-traumatic stress disorder, and generalized anxiety disorder

**DOI:** 10.3389/fpsyt.2022.1020214

**Published:** 2022-12-21

**Authors:** Joshua Willms, Ben McCauley, Lindsay Kerr, Peyton Presto, Ankith Arun, Nazeen Shah, Kierra Irby, Megan Strawn, Jonathan Kopel

**Affiliations:** ^1^Department of Pharmacology and Neuroscience, Texas Tech University Health Sciences Center, Lubbock, TX, United States; ^2^Denovo Therapy, Lubbock, TX, United States; ^3^Researchers for Change, Lubbock, TX, United States; ^4^Department of Cell Biology and Biochemistry, Texas Tech University Health Sciences Center, Lubbock, TX, United States

**Keywords:** ketamine, depression, suicidality, medical school, ketamine assisted psychotherapy

## Abstract

Suicide is the most common cause of death in male resident physicians and the second most common cause of death in resident physicians overall. Physicians also experience high rates of major depressive disorder (MDD), post-traumatic stress disorder (PTSD), and burnout. These conditions frequently develop during medical school, and threaten not only physicians but the patients they care for. A 30-year-old medical student presented to our clinic with a history of treatment-resistant depression (TRD), generalized anxiety disorder (GAD), PTSD, and 5 years of daily suicidal ideation. Previous treatments included therapy, lifestyle modifications, and various combinations of six antidepressants. These interventions had little effect on the patient’s mental health. The patient was treated at our clinic with an 8-month regimen of IV ketamine infusions and ketamine-assisted psychotherapy (KAP). The patient achieved remission from suicidality and PTSD within 1 month; and TRD and GAD within 7 months. The patient’s Patient Health Questionnaire (PHQ-9) score decreased from 25 (severe depression) to 1 (not depressed). These findings suggest that ketamine and KAP may represent effective interventions for mental health applications in healthcare professionals. The patient made the unique decision to attempt to type narrative journals during four of his ketamine infusions (doses ranged from 1.8 to 2.1 mg/kg/h IV). The patient successfully typed detailed journals throughout each 1-h ketamine infusion. To our knowledge, these journals represent the first independently typed, first-person, real-time narratives of ketamine-induced non ordinary states of consciousness. The transcripts of these journals may provide useful insights for clinicians, particularly in the context of KAP.

## Introduction

Medical students and physicians experience high rates of mental health disorders. A meta-analysis of 183 studies found that 27% of medical students experience depression, but only 16% of students with depression sought treatment (1). Suicide is the leading cause of death in male resident physicians, and the fourth leading cause of death in female resident physicians (2). A systematic review of 17 studies reported a 12-month prevalence of suicidal ideation in medical students of 7–36%, and a lifetime prevalence of up to 54% (3). Multiple cross-sectional and longitudinal studies report high rates of anxiety in medical students (4–10). A cross-sectional national survey reported positive PTSD screens in 22% of surgical residents, and that an additional 35% of surgical residents were at risk for PTSD (11). Up to 80% of medical students experience humiliation, belittlement, verbal abuse, or discrimination by their superiors, which can lead to symptoms of post-traumatic stress (12–15). Often attributed to stressors such as high workloads, rigorous study requirements, emotional burdens, and financial strains (1), mental health disorders among medical students remains a critically important healthcare concern due not only to impacts on students’ quality of life but also to potential repercussions on long-term patient care (6).

First line interventions for MDD, PTSD, GAD, and suicidality include SSRIs, SNRIs, tricyclic antidepressants, atypical antidepressants, psychotherapy, and counseling (16–18). Many patients do not respond well to these treatments (16, 17, 19–27). For example, approximately one-third of MDD patients report inadequate remission rates even after multiple treatment attempts and are said to suffer from treatment-resistant depression (28, 29). For individuals with TRD, cognitive-behavioral therapy (CBT) is the most commonly implemented form of psychotherapy (16), though several studies have reported that CBT may be most effective as an adjunct to pharmacotherapy (30, 31). Although electroconvulsive therapy can be effective for TRD, it is often associated with high cost (32) and adverse cognitive effects (33). Therefore, there is an urgent need for improved therapeutic strategies for patients suffering from TRD.

Burgeoning evidence suggests that psychedelic medications may represent breakthrough treatments for numerous mental health disorders (34–36). (R,S)-Ketamine (ketamine), a dissociative anesthetic with psychedelic properties, reduces symptoms of depression, PTSD, and suicidality when administered at subanesthetic doses (25, 37–40). Extensive research has been published on the pharmacokinetics, pharmacodynamics, and cognitive effects of ketamine (40–43). A wide array of subjective rating scales, cognitive tasks, patient interviews, clinical assessments, and brain imaging techniques have been used to study the effects of ketamine on cognition, dissociation, concentration, verbal fluency, motor coordination, mood, memory, and perception during or shortly after ketamine infusions (44–49). Less is known, however, about the subjective experiences of patients during ketamine-induced altered states of consciousness (i.e., emotional processing, “dreams,” meditation, ego dissolution, reliving traumatic experiences, philosophical revelations), in part because these were not traditionally considered to be clinically relevant (34, 50). However, in the context of ketamine-assisted psychotherapy (KAP), clinicians interact with patients who are receiving low-dose ketamine (51–54). In this context, the psychedelic properties of ketamine (i.e., increased receptivity to new ideas, ego dissolution, time-out from ordinary consciousness) represent clinically useful tools, as opposed to problematic side effects (53, 55).

A 30-year-old male medical student was treated for severe depression and suicidality at our clinic with a combination of IV ketamine infusions, KAP, and psychotherapy (Table 1). The patient independently chose to type narrative journals (Supplementary Table 1: Ketamine Journals 1–4) documenting his subjective experiences during four of his normally scheduled ketamine infusions (doses ranged from 1.8 to 2.1 mg/kg IV over 1 h). These journals include detailed descriptions of what he saw, heard, smelled, felt, and thought during ketamine-induced altered states of consciousness, as well as his perceptions of space, time, and self. In this case study, we report the success of an 8-month regimen of ketamine infusions, KAP, and psychotherapy to reduce suicidality, TRD, and PTSD in a medical student. We also share the transcripts of Ketamine Journals 1–4, conduct quantitative and qualitative analysis of the journals, compare independent typing to established methodologies for evaluating patients during ketamine infusions, and explore potential implications for clinicians.

**TABLE 1:** Timeline of diagnoses and pharmacologic interventions.

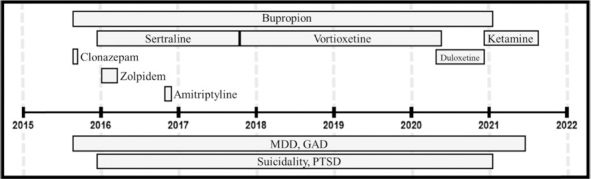						
Diagnosis/ Intervention	Dose	Start	Stop	Weeks	Response	Notes
**MDD**	**NA**	**8/27/2015**	**6/21/2021**	**304**	**NA**	First diagnosed on 8/27/2015 by a psychiatrist at TTPPC Monitored by psychiatrists at TTPPC from 2015 to 2016 Monitored by a psychiatrist at an independent clinic from 2016 to 2021 Monitored via PHQ-9 and mood scores at Denovo from 2020 to 2022 (Figure 1) Remission achieved in June of 2021 (after 8 ketamine infusions and 2 KAP sessions)
**GAD**	**NA**	**8/27/2015**	**6/21/2021**	**304**	**NA**	First diagnosed on 8/27/2015 by a psychiatrist at TTPPC Monitored by psychiatrists at TTPPC from 2015 to 2016 Monitored by a psychiatrist at an independent clinic from 2016 to 2021 Remission achieved in June of 2021 (after 8 ketamine infusions and 2 KAP sessions)
**Bupropion**	**150–300 mg**	**8/27/2015**	**1/12/2021**	**281**	**No improvement**	**Prescribed for depression, anxiety**
	150 mg	8/27/2015	9/9/2015	2	No improvement	Started patient at 150 mg with a plan titrate dose up as needed
	300 mg	9/9/2015	12/9/2015	13	No improvement	Increased dose due to lack of response
	150 mg	12/9/2015	1/8/2016	4	No improvement	Decreased dose due to side effects
	300 mg	1/8/2016	12/19/2016	49	No improvement	Increased dose due to lack of response
	150 mg	12/19/2016	1/27/2017	6	No improvement	Lowered dose due to side effects, then discontinued due to side effects
	150 mg	5/20/2020	1/12/2021	34	No improvement	Restarted, then discontinued again due to side effects
**Clonazepam**	**0.5 mg**	**8/27/2015**	**9/17/2015**	**3**	**Minimal response**	**Prescribed for anxiety; patient stopped taking due to concerns about dependence**
**Suicidality**	**NA**	**Late 2015**	**1/10/2021**	**∼267**	**NA**	First reported in late 2015 to a psychiatrist at TTPPC Acute suicidal intention hidden from psychiatrist during crisis visit at TTPPC on 3/2/2016 due to fear of hospitalization Suicide risk assessed seven times from 3/2/2016 to 9/23/2016 at TTPPC Monitored by an independent psychiatrist from 2016 to 2021 Monitored via PHQ-9 question #9 at Denovo from 2020 to 2022 (Figure 1) Remission achieved in December of 2020 (after first ketamine treatment)
**PTSD**	**NA**	**Late 2015**	**1/10/2021**	**∼267**	**NA**	First reported in late 2015 to a psychiatrist at TTPPC Monitored by an independent psychiatrist from 2016 to 2021 Remission achieved in December of 2020 (after first ketamine treatment)
**Therapy/Behavioral interventions**	**NA**	**9/30/2015**	**2021**	**305**	**Prevented suicide, temporary respite from symptoms**	**Behavioral interventions for the patient included therapy, martial arts, and mentorship**
	Therapy	9/30/2015	9/23/2016	51	No improvement	Patient was seen by a therapist at TTUHSC six times as part of his school’s program of assistance, and four times by a psychiatric physician’s assistant for CBT. Discontinued in favor of focusing on a relationship with a mentor
	Martial arts	2016	2021	∼291	Prevented suicide, temporary respite from symptoms	Patient regularly practiced Brazilian Jiu Jitsu and mixed martial arts. The patient reported that grappling and fighting gave him community, exercise, and temporary mental breaks from depression, anxiety, and suicidality. Training prior to 2016 not reported in this study
	Mentorship	2016	2021	∼291	Prevented suicide	Patient was seen by a retired psychiatrist on a weekly to monthly basis from 2016 to 2021 in a therapeutic and mentoring capacity. The patient reported that these visits prevented him from committing suicide, but did not resolve his depression, anxiety, or suicidal ideation
**Sertraline**	**50–100 mg**	**12/9/2015**	**10/17/2017**	**97**	**No improvement**	**Prescribed for depression and anxiety**
	50 mg	12/9/2015	3/2/2016	12	No improvement	Started patient at 50 mg with a plan to titrate dose up as needed
	75 mg	3/2/2016	3/14/2016	2	No improvement	Increased dose due to lack of response
	100 mg	3/14/2016	10/17/2017	83	No improvement	Increased dose due to lack of response, discontinued due to side effects
**Zolpidem**	**5 mg**	**1/8/2016**	**3/2/2016**	**8**	**Minimal response**	**Prescribed for insomnia; patient stopped taking due to concerns about dependence**
**Vortioxetine**	**10 mg**	**10/17/2017**	**5/20/2020**	**135**	**No improvement**	**Started for depression, discontinued due to lack of response**
**Amitriptyline**	**25 mg**	**10/28/2016**	**11/28/2016**	**4**	**No improvement**	**Prescribed for depression, anxiety; discontinued due to side effects**
**Duloxetine**	**30–60 mg**	**4/29/2020**	**12/10/20**	**32**	**No improvement**	**Prescribed for depression, anxiety**
	30 mg	4/29/2020	10/20/2020	25	No improvement	Started patient at 30 mg with a plan to titrate dose up as needed
	60 mg	10/20/2020	12/10/2020	7	No improvement	Increased dose due to lack of response, discontinued due to side effects
**Ketamine**	**0.9–1.8 mg/kg/h**	**12/10/2020**	**8/18/2021**	**36**	**Full remission of mental health disorders**	**Patient started 8-month treatment regimen consisting of 12 IV ketamine infusions, 2 KAP sessions, and 2 psychotherapy sessions**
#1 (IV)	0.9 mg/kg/h	12/10/2020	NA	NA	Acute improvement	PHQ-9 decreased from 21 prior to treatment to 5 the next time it was measured (1/10/2021). Patient reported resolution of suicidal ideation
#2 (IV)	1.1 mg/kg/h	12/15/2020	NA	NA	Acute improvement	Infusion 2 was administered as soon as possible after the first infusion. Dose was titrated up
#3 (IV)	1.2 mg/kg/h	12/17/2020	NA	NA	Acute improvement	Infusion 3 was administered as soon as possible after the second infusion. Dose was titrated up
#4 (IV)	1.4 mg/kg/h	1/9/2021	NA	NA	Acute improvement	Patient’s schedule delayed Infusion 4. Dose was titrated up
#5 (IV)	1.6 mg/kg/h	2/11/2021	NA	NA	Maintained improvements	Patient’s schedule and financial concerns substantially delayed Infusion 5. PHQ-9 score began to rise during this gap in treatment. Dose titrated up
#6 (IV)	1.4 mg/kg/h	3/6/2021	NA	NA	Maintained improvements	Dose titrated down based on patient feedback and long recovery after Infusion 5
#7 (IV)	1.7 mg/kg/h	4/7/2021	NA	NA	Maintained improvements	Dose titrated up based on patient feedback and scheduling constraints (patient would be unable to receive treatment again in the near future)
#8 (IV)	2.1 mg/kg/h	5/13/2021	NA	NA	Maintained improvements	Dose titrated up based on patient feedback and PHQ-9 score beginning to rise during the gap in treatment between Infusions 7 and 8
#9 (KAP)	0.9 mg/kg	5/29/2021	NA	NA	Maintained improvements	First KAP session. Psychotherapy was integrated with a lower dose of ketamine
#10 (KAP)	1.0 mg/kg	6/17/2021	NA	NA	Maintained improvements	Second KAP session. Psychotherapy was integrated with a lower dose of ketamine. Patient’s PHQ-9 score reached 1 for the first time after this treatment
#11 (IV)	1.2 mg/kg/h	6/26/2021	NA	NA	Maintained improvements	Dose titrated down from last IV infusion based on patient feedback and to mitigate recovery time
#12 (IV)	1.6 mg/kg/h	7/7/2021	NA	NA	Maintained improvements	Dose titrated up based on patient feedback and therapist’s suggestion
#13 (IV)	1.6 mg/kg/h	7/14/2021	NA	NA	Maintained improvements	Dose maintained based on patient feedback and therapist’s suggestion
Psychotherapy	NA	7/16/2021	NA	NA	Maintained improvements	No ketamine administered. Patient met with psychotherapist
#14 (IV)	1.6 mg/kg/h	7/27/2021	NA	NA	Maintained improvements	Dose maintained based on patient feedback and therapist’s suggestion. Patient’s PHQ-9 score reached 1 for the second time after this treatment
Psychotherapy	NA	8/18/2021	NA	NA	Maintained improvements	No ketamine administered. Patient met with psychotherapist

Timing of diagnoses for mental health disorders and timing, dose, and response to medications. Diagnoses and medications were listed in order of when they were first occurred or were prescribed. Responses to medications were based on clinical notes, pharmacy records, and the patient’s recollection when necessary. Bupropion, sertraline, amitriptyline, duloxetine, and vortioxetine were taken orally once per day. Clonazepam was taken orally up to twice daily as needed. Zolpidem was taken orally once per day in the evening as needed. Ketamine infusions were administered via IV over 1 h. Ketamine for KAP sessions was administered via a single intramuscular shot. Medications not relevant to the present study (i.e., ibuprofen) were not included. Rows with medications in bold: indicate dose range and total time period over which the medication was prescribed; sub-rows (not in bold) document timing, response, and rationale for changes in dose. Exact dates were reported when available. Ketamine treatments were labeled 1–14 (comprised of 12 IV infusions and 2 KAP sessions) in order of date. TTPPC, Texas Tech Physicians Psychiatry Clinic.

**FIGURE 1:**
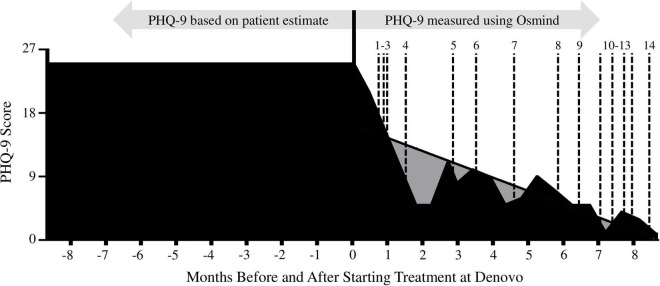
The patient’s Patient Health Questionnaire (PHQ-9) scores 8 months prior to and after starting treatment at Denovo Therapy. PHQ-9 scores prior to establishing care at Denovo Therapy were based on patient estimates, after establishing care scores were recorded in Osmind. Gray shading: represents the area under a simple linear regression of PHQ-9 data from day 1 (first PHQ-9 measurement; measured 3 weeks prior to first ketamine treatment) to day 258 (last PHQ-9 measurement taken during the 8-month treatment regimen). Dotted lines: correspond to ketamine treatment dates (1–14).

## Case report

### Patient information, clinical findings, and timeline

A 30-year-old, 77-kg, Caucasian male medical student presented to Denovo Therapy (Denovo)^1^ with a history of TRD, GAD, PTSD, and 5 years of daily suicidal ideation. The patient reported depressed mood, difficulty concentrating, decreased academic performance, anhedonia, loss of appetite, insomnia, recurrent nightmares, loss of energy, loss of interest in social activities, feelings of worthlessness, grief due to the end of a relationship, and anxiety about the future. The patient perseverated on, fixated on, and re-lived specific traumatic events associated with his medical training. The patient also reported a history of household instability and parental fighting throughout his childhood, and chronic back pain from the ages of approximately 15–30. The patient engaged in heavy binge drinking for 1 month in early 2016 to self-medicate for depression. Over the course of 5 years, the patient was seen by multiple psychiatrists and therapists and was prescribed strategic combinations of seven SSRIs, SNRIs, atypical antidepressants, and benzodiazepines (Table 1). None of these interventions had a meaningful effect on the patient’s mental health. The patient was recommended for electroconvulsive therapy, but refused treatment due to time constraints and lack of proximity to a treatment facility.

### Diagnostic assessment

#### Diagnostic methods

**FIGURE 2:**
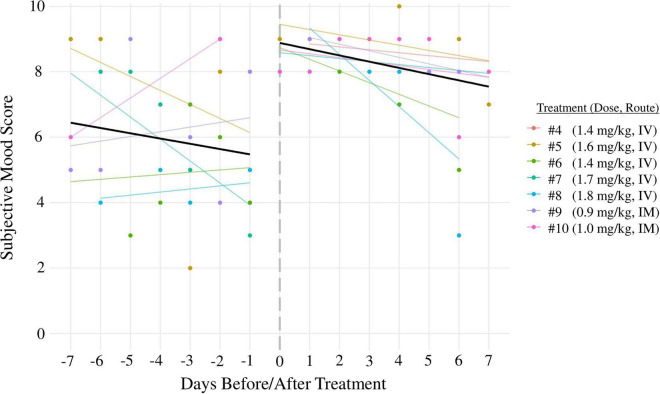
Mood scores 7 days before ketamine treatments (**left side** of graph) were compared to mood scores 7 days after (**right side** of graph) for ketamine treatment sessions 4–10. Black lines: linear regression lines (days before/after treatment predicting mood) averaged across all sessions. Colored lines: linear regression lines for each individual treatment session. All treatments were administered over the course of 1 h. Treatments 1–3 were excluded from mood score analysis because the time between sessions was less than 7 days; treatments 11–14 were excluded because the patient did not document his mood scores regularly during that time period.

Diagnoses for MDD, GAD, PTSD, and suicidality were made using the Structured Clinical Interview for DSM-IV (SCID-I); the Clinician-Administered PTSD Scale for DSM-5 (CAPS-5); and the Columbia-Suicide Severity Rating Scale (C-SSRS) (56–59). TRD diagnosis was made based on inadequate response to multiple antidepressants of different classes taken at adequate doses and durations (Table 1) (60). The Patient Health Questionnaire (PHQ-9), a version of the PRIME-MD diagnostic instrument, was self-administered by the patient using the Osmind electronic health records platform^2^ at regular intervals (Figure 1 and Table 2) (61–64). PHQ-9 was used to monitor depression severity, response to treatments, and for diagnostic purposes. Question #9 of the PHQ-9, “Over the past 2 weeks: Thoughts that you would be better off dead, or thoughts of hurting yourself in some way?” and the follow up to Question #9, “Do you have an active intent or plan to harm yourself?” were used to monitor suicidality (Table 2). Subjective mood scores (scale from 1 to 10, where 1 is worst, and 10 is best) with optional journal entries were recorded by the patient daily in Osmind (Figure 2).

**TABLE 2:** Long-term impact of ketamine and ketamine assisted psychotherapy (KAP) on the patient’s Patient Health Questionnaire (PHQ-9) scores.

Date	Month #	PHQ-9 score	PHQ-9 question #9	Active intent or plan to harm yourself?
**PHQ-9 scores during 8-month regimen**				
11/18/2020	1	25	4	Yes
12/1/2020	1	21	4	Yes
1/10/2021	1	5	1	No
1/24/2021	2	5	1	No
2/7/2021	2	11	2	No
2/14/2021	2	8	2	No
2/28/2021	3	10	2	No
3/14/2021	3	9	1	No
3/27/2021	4	5	1	No
4/11/2021	4	6	1	No
4/24/2021	5	9	1	No
5/9/2021	5	7	2	No
5/23/2021	6	5	1	No
6/7/2021	6	5	1	No
6/21/2021	7	1	1	No
7/5/2021	7	4	1	No
7/19/2021	8	3	1	No
8/2/2021	8	1	1	No
**PHQ-9 scores after 8-month regimen**				
8/30/2021	9	6	1	No
9/18/2021	10	5	1	No
10/2/2021	10	3	1	No
10/16/2021	11	7	1	No
10/31/2021	12	1	1	No
11/17/2021	12	2	1	No
12/4/2021	13	10	1	No
12/27/2021	14	6	1	No
1/10/2022	14	5	1	No
3/2/2022	16	4	1	No
3/22/2022	17	5	1	No
4/19/2022	18	3	1	No
5/5/2022	18	2	1	No
5/19/2022	19	4	1	No

Dates, number of months after starting treatment at Denovo, and PHQ-9 scores during and after the 8-month treatment regimen. Responses to PHQ-9 Question #9 “Over the past 2 weeks: Thoughts that you would be better off dead, or of hurting yourself in some way?” were reported individually (1 = not at all, 2 = several days, 3 = more than half the days, and 4 = nearly every day).

#### Diagnostic challenges

The patient was evaluated at five separate clinics by six psychiatrists, two therapists, and one psychologist prior to intake and treatment at our clinic. The diagnostic tools, reporting methods, and availability of data differed widely from clinic to clinic, limiting the degree to which diagnoses could be compared. The patient reported that he hid the severity of his suicidal ideation from all but one of his mental health providers due to fear of hospitalization; unfortunately this provider did not measure or document the patient’s suicidality over time. The patient’s rigorous academic schedule was a barrier for the use of structured, standardized diagnostic assessments.

#### Diagnostic reasoning

The patient presented to our clinic with prior diagnoses of MDD, GAD, PTSD, and suicidality from psychiatrists at independent clinics. Tools used by these clinicians included SCID-I, CAPS-5, and C-SSRS. Depression severity and response to treatment was monitored by Denovo using the PHQ-9 questionnaire and subjective mood scores. Although the PHQ-9 is less sophisticated than other diagnostic tools for depression (i.e., SCID-I), it is brief, does not require a clinician to administer it, and has been validated as a measure of depression severity and for monitoring treatment outcomes (62–64). The brevity and simplicity of the PHQ-9 made it a pragmatic choice to gather data over a long period in a medical student who faced time constraints. No long-term rating scales for GAD or PTSD were administered at our clinic. However, MDD, GAD, PTSD, and suicidality were evaluated by an independent psychiatrist approximately once every 3 months throughout the duration of this study.

### Therapeutic intervention

The patient was treated at our clinic with a structured regimen of IV ketamine infusions, KAP, and standard psychotherapy sessions for 8 months. In total, the patient received twelve IV ketamine infusions, two KAP sessions, and two psychotherapy sessions (Table 1). The patient’s blood pressure, echocardiogram, pulse, and oxygen saturation were monitored throughout each intravenous treatment and prior to each KAP session. An integrative approach to ketamine therapy was used to calibrate ketamine doses, with the goal for the patient to experience meaningful, mystical experiences, noting that the efficacy of ketamine increases with the existence of a mystical/psychedelic experience (65, 66). All treatments employed a multimodal psychedelic model of care, including patient education, a comfortable setting, music, eye shades, and encouragement to find meaning from the experiences (54, 67–69). The patient was encouraged to integrate elements from therapy sessions.

#### Intervention ketamine infusions

The timing, type of intervention (IV ketamine infusion vs. KAP vs. psychotherapy), and dose for ketamine treatments were determined based on our clinical protocols and the patient’s PHQ-9 scores (Table 1). The patient’s academic schedule, finances, and willingness to participate were also considered. We note that many TRD patients, similar to this case, are unwilling to try psychotherapy for fear that it will not work for them, either because of previous failed attempts or stigma. Like this case, clinically we observe patients are willing to enter care through the infusion model. Once some results are achieved and a therapeutic relationship is established, we are often able to add other psychotherapeutic modalities. Ondansetron (4–8 mg, sublingual or IV) were administered prior to ketamine treatments to mitigate nausea.

While 0.5 mg/kg (IV infusion) over 40 mins is the most commonly used dose of ketamine for mental health applications, it is not necessarily the optimal dose for every patient (65, 66). Based on the patient’s severe depression, acute suicidality, failure to respond to six antidepressants (Table 1), and limited time to devote to treatment, we elected to start him at a slightly higher dose of 0.9 mg/kg (IV infusion) over 60 mins. While this mg/kg dose is higher, it is notable that the duration of the infusion is also higher. Considering the example above, a dose of 0.5 mg/kg for 40 mins is 0.0125 mg/kg/min, while 0.9 mg/kg for 60 mins is 0.015 mg/kg/min. Longer infusion times allow the patient to enter the experience more gradually. For most patient populations, this encourages the patient to learn to navigate the altered state of consciousness building trust and partnership with the medicine, the experience, and themselves. Subsequent treatments allow for dose escalation based on the patient’s physiological and psychological response. Dose escalations require no previous drop in room air SPO2 below 94%, hemodynamic stability, ambulation within <30 mins of treatment, the patient’s ability to retain and relate elements of their experience, and the patient’s reporting of psychological benefits. Our protocols are consistent with other researchers who also found that repeated treatments with dose escalation showed increased efficacy for TRD patients (70, 71).

#### Ketamine-assisted psychotherapy

Including KAP along with two integrative therapy sessions added a depth of understanding to the client’s experience by addressing core issues related to psychological disturbance. In a KAP session an initial intention setting time was used to allow the patient and therapist to prepare for the psychological work to be done along with ketamine. The patient’s intention in his first KAP was “to notice that even if life feels stressful and stuck, its ok, I’m enough.” The therapist acted as a guide encouraging the client toward certain emotional material based on the client’s intentions and expressed desire for change. As the session continued, the client was able to identify his ability to hold two polarities of emotion at once: stress over his career/medical school program, and his knowledge of his self-worth regardless of vocation. One of the benefits of KAP was that the client could notice challenging historical and emotional material along with new emerging states of awareness (72). The therapist encouraged the client to enjoy the break from his ordinary stressors and utilized the idea of “pendulation” (73) to move toward pain points and then out again to his awareness of the ketamine experience.

#### Psychotherapy

To help the continuity of this new state of expanded reflection, two integration psychotherapy sessions were conducted with the same therapist. The therapist integrated the use of EMDR somatic resourcing to extend the effects of the ketamine experience and the client’s new approach to life. Somatic resourcing with bilateral stimulation is a way for the client to have repeated engagement of the ventral vagal state by remembering both external and internal phenomena that bring them comfort and safety. The patient resourced his felt sense of being at the ketamine clinic. The patient recalled the smells of the office, the touch of the blanket, the feeling of being in the reclined chair, and feeling safe in the presence of his providers, which brought him feelings of peace and relaxation. This somatic resourcing process with bilateral stimulation allowed improved access to the ventral vagal state by putting the client in touch with his ability to tolerate negative affect and stress (74). The patient was encouraged to utilize this felt sense of this resource as a tool for when he met challenges around his work as a medical student.

## Independent journaling during ketamine infusions

The patient independently chose to document his subjective experiences during ketamine infusions. The patient established his own methodology: while reclining in a chair before the start of each infusion, he used pillows to position his hands on a keyboard. To make typing easier, the patient chose not to capitalize words, replaced almost all punctuation with the return key, and asked the provider monitoring him to reorient his hands if they strayed out of position. The patient created custom musical playlists with “marker songs,” and transcribed the lyrics to estimate how long after the start of each infusion he was typing certain sentences. The patient’s goals included: describing ketamine-induced altered states of consciousness while experiencing them from a first-person perspective, determining if it was possible to type during ketamine infusions, and determining if he was able to focus his attention on topics discussed with his therapist. Two versions of each journal are included in this report: an unedited version that reflects what the patient was capable of writing during the infusions and a transcript edited by the patient to correct minor errors and clarify text that would have been difficult to interpret (Supplementary Table 1: Ketamine Journals 1–4). The patient was careful not to alter the original meaning in the edited transcript.

## Results

### Long-term impact of ketamine and ketamine-assisted psychotherapy

The patient’s PHQ-9 score decreased from 25 (severe depression) to 1 (not depressed) after 8 months of treatment at our clinic (Figure 1). A simple linear regression was used to test if time in our treatment regimen significantly predicted the patient’s PHQ-9 score over the course of the 8-month treatment regimen. The overall regression was statistically significant [*R*^2^ = 0.6032, *F*(1, 16) = 24.32, *p* = 0.0002]. Therefore, it was found that time in our treatment regimen significantly predicted the patient’s PHQ-9 score. At 8 months after the first treatment, the patient reported no depression, anhedonia, or generalized anxiety. He no longer experienced recurring nightmares or insomnia. He reported increased energy, ability to focus, and motivation. His academic performance improved, and he reported improvements in interpersonal relationships. The patient’s MDD and GAD were considered to be in remission 7–8 months into the treatment regimen based on a PHQ-9 score of 1 and a clinical interview by an independent psychiatrist. Over the following 10 months, the patient maintained an average PHQ-9 score of 4.3 (range 1–10) (Table 2).

The patient was suicidal for 5 years prior to treatment at Denovo (Table 1). This included an active plan to commit suicide for 3 years and one suicide attempt. The patient’s responses to PHQ-9 Question 9, “Over the past 2 weeks: Thoughts that you would be better off dead, or of hurting yourself in some way?” and follow up question “Do you have an active intent or plan to harm yourself?” changed from “nearly every day” and “yes” prior to his first ketamine treatment to “not at all” and “no” immediately after (Table 2). The patient responded “not at all” to PHQ-9 Question 9 on all but four questionnaires over the next 18 months (on those four questionnaires, the patient responded “several days”). The patient never responded “yes” to the question “Do you have an active intent or plan to harm yourself?” over the 18 months following his first ketamine infusion.

The patient was diagnosed with PTSD by a psychiatrist at an independent clinic in 2015 (Table 1). While no consistent, long-term measure for PTSD was administered by Denovo, the patient reported interacting with and resolving specific traumatic events during his first ketamine infusion. After this infusion, the patient reported that he no longer experienced recurrent, involuntary, or distressing memories about the inciting events, no longer experienced recurrent nightmares related to the inciting events, no longer experienced intense psychological distress in response to external cues that reminded him of the inciting events, no longer avoided memories or external reminders related to the inciting events, no longer experienced persistent negative thoughts about himself, no longer experienced a persistent negative emotional state, no longer felt detached from others, and reported reductions in self-destructive behavior and difficulty concentrating. The patient reported that these improvements were maintained over the next 18 months. These findings were confirmed by a psychiatrist at an independent clinic using unstructured clinical interviews (Table 1).

### Short-term impact of ketamine and ketamine-assisted psychotherapy

The short-term effect of ketamine and KAP on the patient’s mood was evaluated using subjective mood scores (0–10, higher scores indicate better mood). The patient’s subjective mood scores the week prior to treatments 4–10 had an average of 5.94 ± 0.35, compared to 8.20 ± 0.20 the week after, indicating that ketamine treatments had an acute positive effect on the patient’s subjective mood (*p* < 0.05; Figure 2). Treatments 1–3 were excluded from mood score analysis because the time between them was less than 7 days; treatments 11–14 were excluded due to insufficient data.

### Journal transcripts and typing analysis

The patient typed 1,195, 578, 410, and 331 words during the infusions for Ketamine Journals 1–4, respectively. The patient’s typing accuracy was calculated by dividing the number of spelling/grammar errors by the total number of words typed during each ketamine infusion. The patient provided a blindfolded typing sample while sober to serve as a positive control (Supplementary Table 1). The patient’s typing accuracy decreased from 100% while fully conscious to approximately 94, 86, 79, and 84% while typing Ketamine Journals 1–4, respectively.

Ketamine Journals 1–4 included detailed descriptions of the patient’s thoughts, feelings, sensations, and perceptions during ketamine emergence at doses from 1.8 to 2.1 mg/kg/h IV. The patient recorded alterations in his visual, auditory, somatosensory, and olfactory senses; attempts to orient himself to time, place, and self; meditation, prayers to a higher power, and repetitive mantras. The patient wrote one poem, transcribed the lyrics of the music he was listening to, and identified his “marker songs.” He wrote occasional remarks directed toward the provider who was monitoring him. The patient stated that typing was extremely difficult during infusions: he was generally unaware of the position of his hands, his hands felt like they moved on a time delay, and sometimes he couldn’t feel his hands at all. Profound thoughts and deep emotions frequently distracted him from typing. Upon returning to an ordinary state of consciousness after the infusions, the patient did not know if he had successfully typed anything–despite having typed hundreds of words–until removing his blindfold and looking at his computer screen.

## Discussion

Established therapies for MDD, suicidality, GAD, and PTSD include SSRIs, SNRIs, tricyclic antidepressants, atypical antidepressants, psychotherapy, and counseling (17, 18), but many patients do not respond well to these interventions (16, 17, 19–27). In particular, the effect of antidepressants on suicidality is complex and age dependent. A recent meta-analysis of randomized controlled trials found that antidepressants actually *increased* risk for suicidality in individuals less than 25 years old and had a neutral effect on individuals 25–64 years old (75). Because most matriculants begin medical school in their early twenties (76), alternatives to traditional antidepressants should be considered for suicidality in medical students.

The positive effects of ketamine were both immediate and long-term for the patient in this report. The patient experienced an acute decrease in depression and suicidal ideation immediately after his first IV ketamine infusion (0.9 mg/kg, administered IV over 1 h), and long-term resolution of his depression and suicidal ideation after 8 months of treatment (Figure 1 and Table 1). These effects are consistent with the broader literature showing that ketamine is an effective intervention for TRD and suicidal ideation (37, 70, 71, 77). Our findings were consistent with a study conducted by Phillips and colleagues in 2019, which found that repeated ketamine infusions were effective to reduce depression in TRD patients (78).

However, our report differs from the established literature regarding the schedule for ketamine treatments, the doses of ketamine used, and the total number of ketamine treatments administered. Most studies on ketamine for mental health applications utilize 0.5 mg/kg of ketamine (IV) over 40 mins (40, 78). For example, Phillips et al., administered six ketamine infusions (0.5 mg/kg over 40 mins) thrice weekly for 2 weeks (78). Patients who responded well to the first six treatments underwent an additional four treatments, for a total of ten. The patient in the present study could not devote 2 weeks to ketamine infusions due to his academic schedule, was severely depressed, and was actively suicidal. Therefore, we chose to modify his treatment schedule to limit interference with school, slightly increase his starting dose of ketamine, and continue maintenance treatments as needed to prevent relapse. After the first treatment, we calibrated subsequent doses of ketamine based on established recommendations (70, 71) and in partnership with the wishes of the patient and his other mental health providers. Our findings suggest that alternative treatment schedules and dosages of ketamine may be effective for patients who are unable to devote weeks to treatment in a single time block.

Ketamine is also emerging as a potential treatment for PTSD, but more research in this area is needed (79–81). One randomized clinical trial found that ketamine rapidly reduced PTSD symptoms (82), and a study on PTSD in burned service members found that individuals who received perioperative ketamine had a lower prevalence of PTSD than those who did not (83). The patient in the present study was diagnosed with PTSD in 2015, and none of the six antidepressants he took mitigated his symptoms (Table 1). He stated that during his ketamine infusions he interacted with and resolved specific traumatic events, and that after infusions he felt a sense of freedom from persistent negative thoughts related to those events. The patient’s PTSD fully resolved by the end of the treatment regimen. These findings provide further reason to investigate ketamine for PTSD.

Limitations of this report include the lack an active placebo control (i.e., midazolam); a lack of consistency in selection, administration, and reporting of diagnostic measurements across five separate clinics prior to intake at Denovo; and a lack of long-term, recurring measures for PTSD and GAD. A structured, standardized clinical interview was not used to verify remission of PTSD. The patient’s rigorous academic schedule often precluded the use of structured, standardized diagnostic assessments. The patient and providers were not blinded to therapeutic interventions. Because the patient elected to delay KAP and psychotherapy until more than 6 months into his treatment regimen (at which point most symptoms had already improved), it was difficult to compare the therapeutic value of IV infusions, KAP, and psychotherapy. Mood scores were used to monitor short-term changes in the patient’s subjective mood (Figure 2), but this metric has not been validated as a reliable tool for research and is susceptible to bias. The translatability of our findings may be limited because the patient underwent years of therapy prior to his first infusion (which may have modified his response), and because the patient showed a unique level of engagement with the treatment regimen (i.e., typing journals during infusions). While the patient did not experience any major adverse effects, he was unable to study or attend school on the days he received treatments, which could present a challenge for some students. The risk for adverse events from low doses of ketamine, while extremely low in a clinical setting, cannot be fully ruled out (84, 85).

Strengths of this report include the large amount of PHQ-9 data gathered at regular intervals before, during, and up to 10 months after the 8-month treatment regimen; the large amount of diagnostic information from multiple independent sources; excellent documentation of antidepressant dose, timing, and response for comparison to ketamine therapy; and the dramatic improvements seen in the patient’s long term mental health.

Ketamine-assisted psychotherapy is an emerging model of care in which providers interact with patients who are experiencing non-ordinary states of consciousness. Unfortunately, relatively little is known about the subjective experiences of patients during psychedelic experiences, limiting the potential for providers to act as guides. To our knowledge, the patient in this study is the first to use real-time typing as a method to report psychedelic experiences. Published methods to document the effects of ketamine include subjective rating scales, cognitive tasks, patient interviews, clinical assessments, brain imaging, and journaling after returning to ordinary consciousness (44–49). Compared to these methods, typing is unique in that it allowed our patient to generate highly detailed, open-ended descriptions of his experiences. Typing is a relatively fast form of communication and allowed the patient to generate large volumes of data per treatment. The patient’s reports were less likely to be influenced by the amnestic effects of ketamine because they were typed in real time, an advantage over methods in which patients are interviewed minutes to days after waking up (86, 87). The patient’s descriptions were also unique in that they arose from what the patient was motivated to report, as opposed to responding to tightly worded rating scales or questionnaires. In this sense, narrative typing provides insight into what matters about non-ordinary states of consciousness from the perspective of the patient, which could be useful information for KAP providers. However, the authors do not recommend that patients attempt to type during ketamine infusions in general, nor do we recommend typing as a modality for further research. Typed narrative descriptions lack the rigor, reproducibility, and comparability of carefully designed rating scales and cognitive tasks. They also require substantial skill, motivation, and effort on the part of the patient. Our patient reported that typing during infusions was extremely difficult, exhausting, and sometimes retracted from his experience.

The experiential effects of ketamine documented by the patient were largely consistent with the established literature. Similar to other reports, the patient experienced dissociative symptoms (47); psychotomimetic effects (38, 39, 88); alterations in hearing, vision, and proprioception (89–91); and impaired cognition, concentration, and memory (44, 46, 49, 88). The patient also experienced decreased motor coordination (49). However, the patient in this study was unique in that he was able to independently maintain directed attention toward a predetermined goal throughout ketamine infusions at doses as high as 2.1 mg/kg/h (IV). Although there is no way to fully communicate psychedelic experiences to those who have never had them, our patient’s detailed, real-time journals may provide helpful insights for providers. Patients who are hesitant to try ketamine therapy may also benefit from reading the experiences of another patient.

Medical students and physicians are a uniquely at-risk population for mental health disorders, and the negative downstream effects of these conditions on their patients and communities cannot be overemphasized (92, 93). Although ketamine and KAP may be expensive and require substantial time investments, they are more affordable than most higher levels of care after first line options have failed. While time constraints for ketamine and KAP are legitimate, our report demonstrates that ketamine treatments can work into a busy schedule where electroconvulsive therapy and in-patient hospitalization would be more difficult. Patients also determine whether ketamine will work for them faster than antidepressants, which require at least a month to take effect. Perhaps most importantly, ketamine rapidly reverses suicidality (37). For this reason alone, the authors argue that ketamine is a reasonable first line consideration for suicidality and other severe mental health indications in medical students. While no treatment modality can replace the need for substantial reform to a healthcare system that contributes to high rates of mental health disorders in medical students and physicians, ketamine and KAP represent previously untapped treatment modalities that could benefit this population.

## Patient perspective

“Traumatic events during my medical training caused me to develop severe depression. I had suicidal thoughts during almost every quiet/non-distracted moment for over 3 years, and I had nightmares almost every night. The constant pressure to perform well in school combined with the rigorous schedule made it nearly impossible for me to find time to grieve, rest, or process my emotions. I was seen by multiple counselors, therapists, and psychiatrists, but my symptoms continued to worsen. Months waiting to see if various iterations of antidepressants would help were months of suffering, and when those treatments failed, I felt hopeless. I began to rationalize ways that I could take my own life but still help people, for example by becoming an organ donor. I decided to try ketamine treatments as a last resort, even though I was highly skeptical that anything could help me. During my first ketamine infusion, I re-experienced and emotionally processed some of the worst traumatic events associated with my training. I told a person who is no longer in my life that I love them, apologized for hurting that person, and forgave myself for past mistakes. Immediately upon waking from this treatment the constant suicidal ideation and self-hatred were gone, like a tumor had been removed from my brain. I was able to sleep peacefully without nightmares, meditate quietly without intrusive thoughts, and reconnect with friends and family who I had been distancing myself from (an attempt to lessen the pain they would feel if I took my own life). I genuinely looked forward to each new day instead of dreading the future, my ability to study greatly improved, and I was also able to process new traumatic experiences without descending into severe depression. Although I experienced mild to moderate depression/anxiety at times over the next year (primarily due to external stressors from school), follow-up treatments with ketamine and psychotherapy prevented me from relapsing anywhere close to my previous state. In my view, the years of cognitive behavioral therapy, counseling, and psychiatric care I underwent prior to ketamine treatments laid the groundwork for improvements to take place; but it was the ketamine that provided the breakthrough necessary to free me from my depression. Before ketamine treatments, I couldn’t imagine what it would be like to want to be alive; after, I couldn’t imagine what it would be like to want to be dead.

I decided to try to write down what was happening while I was “dreaming” during infusions to help others feel less nervous about ketamine therapy. The staff at Denovo was skeptical that I would be able to type during infusions, but I am grateful that they allowed me to attempt to do so. I also wanted to write during my infusions to document the extraordinary things I experienced, test whether I could direct my focus toward topics I had discussed with therapists, explore what the mind is capable of, and decrease the stigma around ketamine. Each time that I typed a journal, I was surprised that I had typed when I woke up. I was surprised to see pages and pages of notes on my computer screen. Over time though, and after reading my notes, my memories from the ketamine “dreams” partially returned, including what it was like to type. Typing was extremely difficult. It was like trying to remember the name of someone you met only once years ago, and you were at the bottom of the ocean with a 2-mile-long stick attached to a pen trying to write that person’s name on a piece of paper on a moving boat–and everything was on a time delay–and you couldn’t feel the stick. I am grateful that I can read my notes to reflect on what my experiences taught me.”

## Data availability statement

The original contributions presented in this study are included in the article/Supplementary material, further inquiries can be directed to the corresponding author.

## Ethics statement

Ethical review and approval was not required for the study on human participants in accordance with the local legislation and institutional requirements. The patients/participants provided their written informed consent to participate in this study. Written informed consent was obtained from the individual for the publication of any potentially identifiable data included in this article.

## Author contributions

JW collected the data, analyzed transcripts, and wrote initial drafts of the abstract and case description. BM conducted the patient intake and facilitated all treatments at Denovo Therapy. LK conducted KAP sessions. AA calculated the dosages of ketamine and conducted a literature review. PP wrote the initial draft of the introduction and assisted with editing. NS assisted with data collection, assisted with the literature review, and contributed to the introduction. KI designed the figures and tables. MS conducted literature review and assisted with editing. BM, LK, and JK provided expertise and advice. All authors contributed to the article and approved the submitted version.

## Footnotes

1
https://denovotherapy.com/

2
www.osmind.com
