# Supplementary material for: Case report: Medical student types journals during ketamine infusions for suicidal ideation, treatment-resistant depression, post-traumatic stress disorder, and generalized anxiety disorder
Source: Front Psychiatry. 2022 Dec 21;13:1020214. doi: 10.3389/fpsyt.2022.1020214 (PMC9811588; doi:10.3389/fpsyt.2022.1020214)
Supplement: Supplementary file 1 [file Table_1.docx]

**Supplementary Table 1. Ketamine Journals.** Notes typed by the patient while sober and during four 1-hour IV ketamine infusions. *Transcript (Raw Text)*: the original text typed by the patient. *Transcript (Edited by Patient)*: the patient fixed spelling and grammatical errors without altering the meaning of the text. *Commentary*: commentary by the patient and our research team. Time markers (underlined, bold) were placed in the commentary column to indicate the approximate time at which text was typed after the start of ketamine infusions.

**Control Typing Sample:** Notes typed by the patient without medication while wearing a blindfold.

| **Transcript (Raw Text)** | **Transcript (Edited by Patient)** | **Commentary** |
| --- | --- | --- |
| *This is an example of how well I can type with a blindfold on. I can type proficiently. I have been typing ever since I was a child. I type every day without looking at the keyboard. I’m fully awake right now; not in an altered state of consciousness or receiving any medication. I cannot see the keyboard, but I can feel the small bump on the “f” and “j” keys to orient.*  *I can type the alphabet:  abcdefghijklmnopqrstuvwxyz  I can also type numbers 1-9:  123456789* | *This is an example of how well I can type with a blindfold on. I can type proficiently. I have been typing ever since I was a child. I type every day without looking at the keyboard. I’m fully awake right now; not in an altered state of consciousness or receiving any medication. I cannot see the keyboard, but I can feel the small bump on the “f” and “j” keys to orient.*  *I can type the alphabet:  abcdefghijklmnopqrstuvwxyz  I can also type numbers 1-9:  123456789* | Patient is fully awake and oriented.  The patient provided a sample of his typing while blindfolded, but not in an altered state of consciousness to serve as a positive control (the patient was also blindfolded during the ketamine infusions).  The patient can type at approximately 72 words per minute with 100% accuracy (medium difficulty test, typingtest.com). |

**Ketamine Journal 1:** Notes typed by the patient during a ketamine infusion (2.1 mg/kg/hr IV).

| **Transcript (Raw Text)** | **Transcript (Edited by Patient)** | **Commentary** |
| --- | --- | --- |
| *OK. Im sittin gin the chair. Fully awake.*  *I’m about to get drug. I feel safe here at the clinic. Everyone is very kind. Already feeling it. I hear a rining i hear the music i can smell the distinct clinic scent that is comforting*  *Wow the drug hit fast i love veryone i want everything to be ok my hands felel a bi odd but i think i can type im already really deep start recrdoing if you can wow This was very fast*  *I hear the music fingers fell odd*  *Peacepeaceful violin veauty \d*  *Just*  *Checking thanks*  *Violin very beautiful. Rolling, beaurtiful*  *Beauftufl*  *So ba*  *So beautiful*  *Falling*  *A plane a safe place a safe place thankful i am calm swirling swirling blue like water deep comfort im ok i feel ok i accept myself looking up towards a light . . uplifting. Swirling. My name i s [name of the patient; repeated twice]*  *Swirling*  *I feel calm relaxed, i can breath*  *Calm*  *Calm*  *Peace*  *Peace*  *Peaceful*  *Its foing to be ok*  *Im going to be ok*  *Im going to be ok*  *I am excited to explore*  *Bbeauty green, a ship, falling, a road, beautiful music, i feel at home, green , a smile, happiness, warmth, it is covering me i feel like a large3 warm blanket . a big blanket! Safe! I feel so safe. And happy . and calm. Thank you*  *I’m glad that i am here*  *Whats next?*  *I see a waterfall*  *A beach*  *Water up and down*  *Beautiful waves*  *I can see a peautuiful painting of the sea*  *Now weverything is changing*  *Amazing*  *Wow*  *A golden beautiful place a treasure trove , like in dnd.*  *Now i see black, i am in space*  *A beautiful sky*  *Purple couds*  *Amazing sky*  *The music is important, and i think the dose is really starting*  *A purple and yellow drip that is very beautiful and flowing, a s in the shape of a cliff,*  *Very beautiful*  *In sap*  *In space again*  *So many stars*  *Peaceful*  *I feel safe*  *I feel like im dreaming*  *The stars are so beautiful at night, they cover they sky, i wish i could see them all, just as much as i love everyone. It is so good to be here . so good to be here on earth with everyone. Everyone, i love all of you. I love you al so sos so so so uch!!!!!*  *I am experiencing beauty.*  *In small things*  *In small details and places*  *I want everyone to be ok*  *I want everyone to be ok*  *Now*  *Are you made of love energy? That is what [name of the provider] just said to me. Are you made of love?*  *I love everyone. I want everyone to be ok.*  *I want everyone to be safe.*  *I see the room! I am in the place that is very deep.*  *Purple clouds, i feel safe, i want to go deeper into this experience. I see indescribable beauty*  *Green, glowing, impact, massive, interstellar, galaxy,*  *I feel safe*  *Letting go for a moment*  *Space*  *We are safe here*  *We are safe here right now*  *Piano started.*  *I’m going to take a break.*  *Still here,*  *Just*  *Floating underwater*  *Now falling through space*  *Millions of little pieces falling all around*  *Wow*  *New music*  *Waves, raves, amazing colors, , so cool,*  *Now is see a road i wonder where we are going?*  *Beautiful musicv*  *Wow*  *Indescribable*  *Flying*  *Flying*  *Ive veen here vefre*  *Ive veen here before*  *Im in space again*  *Just heard the machine.*  *Feels like im in myst*  *A foggy area*  *Mystery*  *[name of significant other] i love you*  *[names of family and friends], i love you*  *Now the world is spinning*  *Exploring a n amazing canyon like in slime rancher*  *Now blank*  *Beauty*  *Beauty*  *Profound bo*  *Profound beauty*  *I love everyone*  *I love everyone so much*  *How is it ithat this thsequences of movemenetns meanstloves*  *I love everyone*  *I want everyone to be ok*  *Please*  *Everyone*  *B e*  *O*  *K*  *Be ok*  *I love you all*  *I love you so much*  *Now waves are going by*  *Peace*  *Who am i*  *I am*  *I*  *Am*  *A crazy golden landscape with*  *Too much to describe*  *So beautiful*  *I love you [name of significant other]*  *.*  *A hallucination is very interesting . like in that movie.*  *[name of provider] said are you divine*  *I feel safe*  *Beautiful*  *Safe*  *Safe*  *Safe*  *Safe*  *Happy*  *Happy*  *I am so thankful*  *Thank*  *I wan ti want to talk to god. Why does everyone nhurt?why ?*  *Please let everyone brbe ok.*  *A an expansive landscape*  *Is before meits beautiful*  *Im safe*  *Peac*  *Peace*  *I love everuyone*  *I love everyone*  *I love everyone*  *A river of peace*  *Who am i ?*  *I see my life*  *I see memories*  *I see happiness*  *I am ok*  *I see the sky*  *I can see the stars*  *The sars@beaty!!!!!!!!room*  *Help everyone*  *Beauty*  *I want to helpeveryone i want to help everyone i want to help everyone*  *What a beautiful place*  *Im in the room*  *I can see everything . i can talk to god. I can look at the unicverse.*  *Beauty*  *I see beauty*  *But i still know who i am.*  *My senses are twisting k can see a whole world of possibilitli3s*  *Beauty*  *Beauty*  *Wow*  *Wow!*  *[name of significant other],, i love you*  *Im very deeo in the experience*  *jj………..*  *I wan t every9one to be ok . love*  *Love*  *Love.*  *Love*  *Love*  *Love*  *Love*  *A new day*  *Risin gu to the sky*  *Beautiful dky*  *Like*  *I see*  *Wow*  *Lim flying*  *K*  *Leace*  *Leace*  *I am still here*  *Here*  *Ere*  *I am ok.*  *Wow*  *So beautiful*  *Im ok*  *This is good music.*  *Where am aI?*  *I know*  *Violin*  *Beautiful*  *Wow*  *I am*  *I am*  *I*  *Screen*  *Screen*  *This experience is is sad sad. The music is sad now. I still know i am me. [first name of the patient]. [last name of the patient; repeated twice]. Jmmmmmmmmmmmmmmm.thank you [name of provider]*  *I am ok.*  *I am just listening to the music now, i think i’m awake ta little bi? No . still in bed. I want everyone to be ok. I want everyone to be ok.*  *The words keep jumping and skipping away from fme*  *I hop ei can feel better soon*  *Ok.*  *I think im wakin gup some*  *No, still u nder*  *Still under.*  *I can see blue skies with red clouds.*  *I think i’m starting to wake up.*  *I think im waking up.*  *I can feel my hands on they keyboard.*  *I w*  *I wonder what the heck just happened.*  *Yah, def waking up now. OK. I’m here, I’m ok. It’*  *It’s interesting that what i wanted to point out is that im here.*  *I think i can only get so deep when i try to communicate what is happening back to the … um… “physical” world. Haha. Ok. Time to wake up i think. Still a little bit woozy, not ready to stand or anything. I can open my eyes and see, but the room is moving all over. I’m waking up.*  *Totally awake. HELLO!*  *I wonder what I typed?*  *eIt was a very interesting experience. I was aware that I was here, but only in the very very back of my mind.*  *I’m a good person who wants to help people.* | *OK. I’m sitting in the chair. Fully awake.*  *I’m about to get drug. I feel safe here at the clinic. Everyone is very kind. Already feeling it. I hear a ringing I hear the music I can smell the distinct clinic scent that is comforting*  *Wow the drug hit fast I love everyone I want everything to be ok my hands feel a bit odd but I think I can type I’m already really deep start recording if you can wow This was very fast*  *I hear the music fingers feel odd*  *Peace peaceful violin beauty*  *Just*  *Checking thanks*  *Violin very beautiful. Rolling, beautiful*  *Beautiful*  *So beautiful*  *So beautiful*  *Falling*  *A place a safe place a safe place thankful I am calm swirling swirling blue like water deep comfort I’m ok I feel ok. I accept myself. Looking up towards a light. Uplifting. Swirling. My name is [name of the patient; repeated twice]*  *Swirling*  *I feel calm relaxed, I can breathe*  *Calm*  *Calm*  *Peace*  *Peace*  *Peaceful*  *It’s going to be ok*  *I’m going to be ok*  *I’m going to be ok*  *I am excited to explore*  *Beauty green, a ship, falling, a road, beautiful music, I feel at home, green, a smile, happiness, warmth, it is covering me I feel like a large warm blanket. A big blanket! Safe! I feel so safe. And happy. and calm. Thank you*  *I’m glad that I am here*  *What’s next?*  *I see a waterfall*  *A beach*  *Water up and down*  *Beautiful waves*  *I can see a beautiful painting of the sea*  *Now everything is changing*  *Amazing*  *Wow*  *A golden beautiful place a treasure trove, like in dnd.*  *Now I see black, I am in space*  *A beautiful sky*  *Purple clouds*  *Amazing sky*  *The music is important, and I think the dose is really starting*  *A purple and yellow drip that is very beautiful and flowing, as in the shape of a cliff,*  *Very beautiful*  *In space*  *In space again*  *So many stars*  *Peaceful*  *I feel safe*  *I feel like I’m dreaming*  *The stars are so beautiful at night, they cover they sky, I wish I could see them all, just as much as I love everyone. It is so good to be here. so good to be here on earth with everyone. Everyone, I love all of you. I love you all so so so so so much!!!!!*  *I am experiencing beauty.*  *In small things*  *In small details and places*  *I want everyone to be ok*  *I want everyone to be ok*  *Now*  *“Are you made of love energy?” That is what [name of the provider] just said to me. Are you made of love?*  *I love everyone. I want everyone to be ok.*  *I want everyone to be safe.*  *I see the room! I am in the place that is very deep.*  *Purple clouds, I feel safe, I want to go deeper into this experience. I see indescribable beauty*  *Green, glowing, impact, massive, interstellar, galaxy,*  *I feel safe*  *Letting go for a moment*  *Space*  *We are safe here*  *We are safe here right now*  *Piano started.*  *I’m going to take a break.*  *Still here,*  *Just*  *Floating underwater*  *Now falling through space*  *Millions of little pieces falling all around*  *Wow*  *New music*  *Waves, raves, amazing colors, so cool,*  *Now I see a road I wonder where we are going?*  *Beautiful music*  *Wow*  *Indescribable*  *Flying*  *Flying*  *I’ve been here before*  *I’ve been here before*  *I’m in space again*  *Just heard the machine.*  *Feels like I’m in Myst*  *A foggy area*  *Mystery*  *[name of significant other] I love you*  *[names of family and friends], I love you*  *Now the world is spinning*  *Exploring an amazing canyon like in Slime Rancher*  *Now blank*  *Beauty*  *Beauty*  *Profound beauty*  *Profound beauty*  *I love everyone*  *I love everyone so much*  *How is it that these sequences of movements mean love*  *I love everyone*  *I want everyone to be ok*  *Please*  *Everyone*  *Be*  *O*  *K*  *Be ok*  *I love you all*  *I love you so much*  *Now waves are going by*  *Peace*  *Who am I*  *I am*  *I*  *Am*  *A crazy golden landscape with*  *Too much to describe*  *So beautiful*  *I love you [name of significant other]*  *A hallucination is very interesting. Like in that movie.*  *[name of provider] said “are you divine?”*  *I feel safe*  *Beautiful*  *Safe*  *Safe*  *Safe*  *Safe*  *Happy*  *Happy*  *I am so thankful*  *Thank*  *I want to talk to God. Why does everyone hurt? Why?*  *Please let everyone be ok.*  *An expansive landscape*  *Is before me it’s beautiful*  *I’m safe*  *Peace*  *Peace*  *I love everyone*  *I love everyone*  *I love everyone*  *A river of peace*  *Who am I?*  *I see my life*  *I see memories*  *I see happiness*  *I am ok*  *I see the sky*  *I can see the stars*  *The stars are beauty!!!!!!!! Room*  *Help everyone*  *Beauty*  *I want to help everyone I want to help everyone I want to help everyone*  *What a beautiful place*  *I’m in the room*  *I can see everything. I can talk to God. I can look at the universe.*  *Beauty*  *I see beauty*  *But I still know who I am.*  *My senses are twisting I can see a whole world of possibilities*  *Beauty*  *Beauty*  *Wow*  *Wow!*  *[name of significant other],, I love you*  *I’m very deep in the experience*  [finding keyboard orientation]  *I want everyone to be ok. Love*  *Love*  *Love*  *Love*  *Love*  *Love*  *Love*  *A new day*  *Rising up to the sky*  *Beautiful day*  *Like*  *I see*  *Wow*  *I’m flying*  *K*  *Peace*  *Peace*  *I am still here*  *Here*  *Here*  *I am ok.*  *Wow*  *So beautiful*  *I’m ok*  *This is good music.*  *Where am I?*  *I know*  *Violin*  *Beautiful*  *Wow*  *I am*  *I am*  *I*  *Screen*  *Screen*  *This experience is is sad sad. The music is sad now. I still know I am me. [first name of the patient]. [last name of the patient; repeated twice].*  [finding keyboard orientation]*. Thank you [name of provider]*  *I am ok.*  *I am just listening to the music now, I think I’m awake a little bit? No. Still in bed. I want everyone to be ok. I want everyone to be ok.*  *The words keep jumping and skipping away from me*  *I hope I can feel better soon*  *Ok.*  *I think I’m waking up some*  *No, still under*  *Still under.*  *I can see blue skies with red clouds.*  *I think I’m starting to wake up.*  *I think I’m waking up.*  *I can feel my hands on the keyboard.*  *I wonder*  *I wonder what the heck just happened.*  *Yah, def waking up now. OK. I’m here, I’m ok. It’s*  *It’s interesting that what I wanted to point out is that I’m here.*  *I think I can only get so deep when I try to communicate what is happening back to the… um… “physical” world. Haha. Ok. Time to wake up I think. Still a little bit woozy, not ready to stand or anything. I can open my eyes and see, but the room is moving all over. I’m waking up.*  *Totally awake. HELLO!*  *I wonder what I typed?*  *It was a very interesting experience. I was aware that I was here, but only in the very very back of my mind.*  *I’m a good person who wants to help people.* | Patient is fully awake and oriented.  **0 Minutes**  Ketamine infusion initiated (2.1 mg/kg administered IV over 1 hour).  The patient requested that we record video so that he could determine whether he was able to type.  Violin music was playing.  The patient asked the provider to check if his hands were on the keyboard.  **10 Minutes**  “dnd” refers to Dungeons & Dragons, a role-playing tabletop game (Wizards of the Coast, 1997).  The patient reported that he was consciously attempting to write a poem; starting from “I am experiencing beauty” to “I want everyone to be ok”.  **20 Minutes**  The patient asked the provider to ask “are you made of love energy” prior to the infusion. The patient heard the provider say those words, transcribed them accurately, and recalled the provider’s name.  According to the patient, “the room” is not the physical room at the clinic, but rather a 3D geometric space he frequently experiences in his mind midway through ketamine infusions. He reported that he often “prays” when he experiences this place.  The patient typed “letting go for a moment” to indicate to himself that he was taking a break from typing. He resumed typing soon after.  The patient took another break from typing. “Still here” was partly to indicate that he still intended to type.  The music playing changed.  **30 Minutes**  “The machine” is the IV infusion pump.  “Myst” is a mystery-solving computer game (Cyan Ventures, 1994).  The patient accurately recalled the names of loved ones and expressed feelings for them.  “Slime Rancher” is an adventure video game (Monomi Park, 2017).  The patient reported that patterns he was making with his hands corresponded to the concept of love.  The patient added a space between “O” and “K” to express emphasis.  The patient reported that the words “I am” held both religious and philosophical significance for him. He wrote “I am” to indicate a sense of timelessness, divinity, and eternity.  The patient correctly recalled and expressed feelings for his significant other.  The movie title that the patient could not recall was “*Inception*” (Nolan, 2010).  The patient asked the provider to ask “are you divine” prior to the infusion. The patient heard the provider say those words, transcribed them accurately, and recalled the provider’s name.  **40 Minutes**  The patient grew up in a Judeo-Christian background and reported praying during the infusion. He reported attempting to process a complex philosophical topic, the problem of evil.  As described previously, “the room” is a 3D geometric space that the patient frequently experiences in his mind midway through ketamine infusions. He reported that “Help everyone” was a prayer request to God.  The patient accurately recalled the name of his significant other.  “*jj………..*” was not an attempt to type words, but rather an attempt by the patient to reorient his hands on the keyboard.  **50 Minutes**  Violin music was playing at this time.  As described previously “I am” is a  phrase that carries philosophical and religious meaning for the patient.  “Screen” is another term that carries philosophical and religious meaning for the patient: it refers to a “movie screen” as an allegory for first-person experiences of reality.  The patient asked the provider to help him keep his hands oriented on the keyboard. “Jmmmmmmm…” was an attempt to find the keys. “Thank you” was directed toward the provider to express appreciation for helping him place his fingers on the correct keys.  **60 Minutes**  The ketamine infusion ended.  The patient begins to return to a normal state of consciousness.  The patient has almost fully returned to a normal state of consciousness and is orienting himself to time and place.  The patient reported being unaware that he had typed during the infusion (at this point he still had his blindfold on and could not see his computer screen). |

**Ketamine Journal 2.** Notes typed by the patient during a ketamine infusion (1.8 mg/kg/hr IV).

| **Transcript (Raw Text)** | **Transcript (Edited by Patient)** | **Commentary** |
| --- | --- | --- |
| *I want everyone to be ok*  *This I s a really intense experiences*  *Room is spinning but not in the ssense that you thingk.*  *Bee s. stay*  *I asked for co*  *cinnomonCCOCCINNdcinomon cinnnnoinininininin*  *I like that*  *Coffee*  *E*  *Wow*  *Wow*  *I asked fr tahe coffeeee*  *It was BOMB wowowowowowoowwowowowowooow*  *Just so fascinatings. I said it and typed it. This is so cool. A ay to study tothey mind. Which which is a way to study the soul*  *I see*  *I feel so good and happy and clear and alright.*  *Peace*  *Its just peace*  *That’s what ive been searchin for*  *Peace*  *Stark change in music*  *Hypnotized*  *Hold tight*  *Hold tight*  *Hold tight*  *Driping lies*  *Paint the slkies*  *All because of you*  *Driping*  *Paint the skieds*  *Only you*  *You make mo*  *You make me move*  *Its all gonna be ok*  *This gives me perspective*  *Can I try cinnomin?*  *Thanks*  *So happy lkjdflskdjflsdkjfsl*  *Calack*  *Cklack*  *Clack*  *Caclack, calc,lacc,lack*  *Everything is going to be ok.*  *Its so ieastyto adoresyou your liekwhat a ba*  *What aa beautiful life*  *What a beautiful filfe*  *What a beautriful life*  *What a beautiful life.*  *Found*  *F*  *Wow*  *Eveyre*  *I foudit*  *Im here*  *Wer*  *We have everyhi*  *We can give this to eveyroen*  *Happpi ess*  *The way I felt*  *Its theostimportant things*  *This*  *Is*  *It*  *I*  *Found*  *It*  *Found it*  *This is the correct dose and also correcr music, ut with timing differences*  *Now im at the last stay by beeber*  *Beet just dropped*  *-09-09-9-09\\*  *I found what I was searching for. Bridged the gap. Still bery much under. Not all the way though.*  *Room spinning*  *Smells were extremely coooool. And*  *This playlist was good*  *I can fell*  *I can feeeel myself starting to return to normal a it*  *The music was good this time*  *I’m still just a bit under ut don’t see thingsthings . the music is good. I need to make playlists for different kinds of people.*  *Waking up a bit more, ut very woozy.*  *F*  *Pretty much conscious now, at least that my ind is clear. There is a distinction there… the point where I know that I’m a k to normal. The aw*  *Theself mon*  *The self monitoring capacity of it. I can monitr my own consciousness. Like assessss my own self.* | *I want everyone to be ok*  *This is a really intense experience*  *Room is spinning but not in the sense that you think.*  *[Justin] Bieber. “Stay”*  *I asked for cinnamon*  *Cinnamon!! CINNAMON!!! Cinnamon!!*  *I like that*  *Coffee*  *Coffee*  *Wow*  *Wow*  *I asked for the coffee!!*  *It was BOMB wowowowowowoowwowowowowooow*  *Just so fascinating. I said it and typed it. This is so cool. A way to study the mind. Which is a way to study the soul*  *I see*  *I feel so good and happy and clear and alright.*  *Peace*  *It’s just peace*  *That’s what I’ve been searching for*  *Peace*  *Stark change in music*  *“Hypnotized*  *Hold tight*  *Hold tight*  *Hold tight*  *Dripping lies*  *Paint the skies*  *All because of you*  *Dripping lies*  *Paint the skies*  *Only you*  *You make move*  *You make me move”*  *It’s all gonna be ok*  *This gives me perspective*  *Can I try cinnamon?*  *Thanks*  *So happy lkjdflskdjflsdkjfsl*  *Clack*  *Clack*  *Clack*  *Clack, clac, clac, clac*  *Everything is going to be ok.*  *“It’s so easy to adore you your like…*  *What a beautiful life*  *What a beautiful life*  *What a beautiful life*  *What a beautiful life*  *What a beautiful life.”*  *Found*  *Found*  *Wow*  *Everything*  *I found it*  *I’m here*  *Where*  *We have everything*  *We can give this to everyone*  *Happiness*  *The way I felt*  *It’s the most important things*  *This*  *Is*  *It*  *I*  *Found*  *It*  *Found it*  *This is the correct dose and also correct music, but with timing differences*  *Now I’m at the last “Stay” by beeber*  *Beet just dropped*  *-09-09-9-09\\*  *I found what I was searching for. Bridged the gap. Still very much under. Not all the way though.*  *Room spinning*  *Smells were extremely coooool. And*  *This playlist was good*  *I can feel*  *I can feel myself starting to return to normal*  *The music was good this time*  *I’m still just a bit under but don’t see things things. The music is good. I need to make playlists for different kinds of people.*  *Waking up a bit more, but very woozy.*  *F*  *Pretty much conscious now, at least that my mind is clear. There is a distinction there… the point where I know that I’m back to normal. The aw*  *The self monitoring*  *The self monitoring capacity of it. I can monitor my own consciousness. Like*  *assess my own self.* | Patient is fully awake and oriented.  **0 Minutes**  Ketamine infusion initiated (1.8 mg/kg administered IV over 1 hour).  **10 Minutes**  **20 Minutes**  As discussed in Journal 1, “the room” is a 3D geometric space that the patient frequently experiences in his mind midway through ketamine infusions.  The patient correctly identified that he was listening to the song “*Stay”*, by Justin Bieber and The Kid LAROI (Bieber, Howard, 2021).  The patient asked the provider to hold up a stick of cinnamon for him to smell periodically during the infusion. “I asked for cinnamon” corresponds to the patient vocalizing a request for cinnamon. The patient then asked the provider to hold a cup of coffee close to his nose.  The patient accurately transcribed 10 lines of “*Make Me Move*”, by Culture Code, featuring KARRA (Bains & Norton, 2016).  The patient vocalized a request to smell cinnamon again and thanked the provider.  The patient decided to loudly type on the keyboard and wrote down the sounds of the keys “clacking”.  **30 Minutes**  The patient accurately transcribed 6 lines of “*Easy to Adore You*”, by Mauve (Stilwell & Eliot, 2019).  The patient reflected on the dose of ketamine and the choice of music.  **40 Minutes**  The patient made his own playlist to listen to during each infusion. He chose to play “*Stay”*, by Justin Bieber and The Kid LAROI (Bieber, Howard, 2021), at three time points during the infusion to help him determine how long he had been in a non-ordinary state of consciousness.  **50 Minutes**  The patient stopped typing for approximately ten minutes.  **60 Minutes**  The ketamine infusion ended.  In this instance, the “room” the patient is referring to the actual room at the clinic.  The patient slowly returned to a normal state of consciousness and oriented himself to time and place.  The patient attempts to discern a distinct point at which he knew he was “back to normal.” |

**Ketamine Journal 3.** Notes typed by the patient during a ketamine infusion (2.0 mg/kg/hr IV).

| **Transcript (Raw Text)** | **Transcript (Edited by Patient)** | **Commentary** |
| --- | --- | --- |
| *K*  *I want to be ok*  *Its ok ‘chai*  *Ok lets spin*  *Memoriesgrid*  *Three d grid glowing*  *Schierke bfro, f*  *Berserek*  *Lovelye*  *A lake*  *Lsdkfj*  *Hi hi*  *I can see the ski*  *Relax*  *Its ok*  *Turn down into experience*  *Hi [name of provider]*  *[name of provider] I h1 thank you .*  *Gonnaa gl*  *Just listen for a little bit happy*  *Its ok1 the music is good*  *Beautiful clouds 🡨 seeing them floating like a wedge, then seeing them floating around and seeing myself asa cloud.*  *Can definitely tell*  *That as the kamaihamaiaiaiiaiaiaiiaiiaiaiaiaia goku*  *Of feelings*  *A kamakha*  *Cant spell that now*  *Haha*  *Why*  *Ok why am I sad*  *Situation*  *How can I react*  *Im ok*  *Hi [name of provider]*  *Backto it.*  *Inverted s\axi*  *Axsis*  *Going to just chill for a bit*  *Ro.*  *Ro..i g*  *Wa e*  *Hi [name of provider; misspelled this time]*  *Both old and new memoreieas are accessb les to me*  *Still know tha tim here*  *D*  *I lo*  *I love you*  *I love you*  *I love you*  *Its not aotut me*  *I wa nt evrye9neoena; eto ge ok*  *Jat the justin beiber part haha*  *I wan tt everyeone stheo ve oke .;*  *laddloh oh I be gukiu pi fou*  *i do the l xame laeli coulee*  *lsdj ;aslkdjf;asdladldlldliasd l;fkajslkdfalsdklsdsldlsdjflkasdjfaje; oa;efawejaoweowoawejoawejoweij owieweadlsldlsdolsdl sdlasdlasdladsl*  *i need ;you to stay lstay*  *I neeed you to stay l*  *Mi just want everyeo eaenlto be ok*  *Thera ees a brautuiful; meacuowi love oyo u*  *Itsa anamaina;sd infa;sdi fa;dij*  *[name of provider] hi [name of provider]*  *I ho pe o o area o e*  *Lie aej eaefjalsiejal;sdfalefl etlseroemasdlsdoieruulsjfowfw] djdjfkjfe;fj*  *Kdddddd*  *Hi*  *‘’*  *D’dsvkk*  *There is a green reoom swirling s g sdlkfja;lsdkjf;alsdjfaldk*  *Doin gone o f thosesszoom oin to the area*  *I I want to wake carea a eo f f aoe afpe ‘a ekimin a really deep pl avaeae’j*  *Ffhd;sdlka sd;lfasdlnsaldfdsfjldsfalsdaslsadja;lsdlas dj;lsim in a really deep staee’rose’constraxdst’*  *I am ‘ wwhtat a ;tdiowa is eman tso ge a lvivls vjaejac’ecjapejalvjxz;cvjzx;lcv z;lxckjvz;lxcjvlzxcvlzxcx*  *I love you*  *oiuHi thereope you havre a good experience . find peace. Thank you.*  *[name of provider]. hi*  *Need to be able to say no, set boundaries, take care of myself* | *K*  *I want to be ok*  *It’s ok ‘chai*  *Ok let’s spin*  *Memories grid*  *3D grid glowing*  *Schierke*  *Berserk*  *Lovely*  *A lake*  *[uncertain]*  *Hi hi*  *I can see the sky*  *Relax*  *Its ok*  *Turn down into [the] experience*  *Hi [name of provider]*  *[name of provider] I h1 thank you.*  *Going to go*  *Just listen for a little bit happy*  *It’s ok the music is good*  *Beautiful clouds 🡨 seeing them floating like a wedge, then seeing them floating around and seeing myself as a cloud.*  *Can definitely tell*  *That as the*  *“Kammeehhaammeehhaa!!” (from Goku)*  *Of feelings*  *A “kamakha”*  *Can’t spell that now*  *Haha*  *Why*  *Ok why am I sad*  *Situation*  *How can I react*  *I’m ok*  *Hi [name of provider]*  *Back to it.*  *Inverted axis*  *Axis*  *Going to just chill for a bit*  *Ro.*  *Ro..i g*  *Wa e*  *Hi [name of provider; misspelled this time]*  *Both old and new memories are accessible to me*  *Still know that I’m here*  *D*  *I love you*  *I love you*  *I love you*  *I love you*  *It’s not about me*  *I want everyone to be ok*  *At the Justin Bieber part haha*  *I want everyone to be ok.*  *“Oh-whoa, oh-whoa, I’ll be fucked up, if you [can’t be right here]*  *I do the same thing…*  *[typing as fast as possible]*  *I need you to stay*  *I need you to stay”*  *I just want everyone to be ok*  *There is a beautiful; [uncertain] I love*  *you*  *It’s a [uncertain]*  *[name of provider] hi [name of provider]*  *I hope you are ok*  *Kdddddd*  *Hi*  *There is a green room swirling*  *[uncertain]*  *Doing some of those zoom in to the area*  *I want to take care of [uncertain] ‘a*  *Ok I’m in a really deep place*  *[uncertain]*  *I’m in a really deep*  *Stage ‘rose’ contrast’*  *I am ‘what a tdiowa is man to be a-*  *live [uncertain]*  *I love you.*  *“Oh hi there hope you had a good experience. Find peace.” Thank you.*  *[name of provider]. Hi*  *Need to be able to say no, set boundaries, take care of myself* | Patient is fully awake and oriented.  **0 Minutes**  Ketamine infusion initiated (2.0 mg/kg administered IV over 1 hour).  **10 Minutes**  The patient typed “K” to check that his finger could press the keys.  The patient is referring to the character Schierke from the manga, *Berserk* (Miura 1989-present).  The patient vocalized a greeting and recognition of the provider.  According to the patient, “Going to go” was a way to say goodbye to the provider as he directed his attention towards his inward experience.  **20 Minutes**  The patient was attempting to communicate the intensity of the emotions he was feeling by comparing it to the voice acting of Sean Schemmel when he yells “Kamehameha!” in the TV show “*Dragon Ball Z*” (Nishio & Yamauchi, 1989-1996).  The patient vocalized a greeting and acknowledgement to the provider, then refocused on his internal experience.  **30 Minutes**  The patient vocalized a greeting and acknowledgement to the provider.  The patient stated that he formed new memories during the infusion that he recalled later during the same infusion. He also recalled and reflected on old memories from earlier in his life.  **40 Minutes**  The patient attempted to transcribe the lyrics of “*Stay”*, by Justin Bieber and The Kid LAROI (Bieber, Howard, 2021), but the tempo was too fast for him to keep up.  The patient vocalized a greeting and checked on the provider.  The patient stated that his hands may have become disoriented on the keyboard.  The patient hit the keys next to the F and J keys (which have bumps) to re-orient his hands.  **50 Minutes**  The patient reported instances of “zooming in” on an object.  The patient stated that he was “very deep in the experience” when he typed these lines, and that he was attempting to type as much as he could primarily to determine if he was capable of typing.  **60 Minutes**  The ketamine infusion ended.  “Oh hi there hope you had a good experience. Find peace.” was stated by the provider when the patient pulled off his eye mask for a moment He typed “thank you” in response.  The patient slowly returned to a normal state of consciousness and oriented himself to time and place.  The patient greeted the provider and reflected on how to develop healthier relationships. |

**Ketamine Journal 4.** Notes typed by the patient during a ketamine infusion (2.0 mg/kg/hr IV).

| **Transcript (Raw Text)** | **Transcript (Edited by Patient)** | **Commentary** |
| --- | --- | --- |
| *Zxcvxc*  *[name of the patient]*  *Kkkkkdddd*  *Laksdjfl;asdkjf*  *I can see the imagesQQQ*  *1*  *Kkjkjkj*  *i see the [microscopy] images I took in three d*  *Ok*  *Set things aside*  *This is just abotume now work through mmy pain*  *This is good for them to*  *So I m ok*  *That sip*  *You get thiem to the rim, then do a sip of a scent and line that up with a uplifting music sound and that sgood*  *Took video*  *Im going to go into the experience*  *I can do [3D imaging analysis] in my mind*  *The more I engage my oniousness*  *The lesss I am able to engage the epereince*  *The more I engagea*  *The ore I kinea wake /*  *Nice*  *I see a beautiful sky*  *That means I im twent;y minutes in*  *Interstesllar music is good for this*  *In wonder if pre gaem*  *Watching beautiful scenery*  *If tha ti nfinflue nces that eperince in a positive way*  *A realization song*  *Could be helpfl fo patnatein ets*  *A aha moment*  *To go*  *To give some one*  *Some to give someneo that*  *Turning*  *Points*  *In their*  *T*  *Life*  *Get them*  *I*  *A different trajectory*  *Feels likfe*  *Feelijust the beaa*  *Just the beat inside*  *My soul the*  *Take me to the other side*  *I k now*  *Catch me like I thtsdo o r odi e*  *Take me*  *To the other side*  *Breaking the futht*  *Wall*  *At the end o f the day I wan tto*  *Help people*  *And now im back to the [microscopy]*  *Espore the universe*  *The muscic is really good*  *Catch me like its do or die*  *I can see*  *[name of the provider; misspelled] I want you to br ok*  *Im gonna go exp*  *Explore*  *Kdkdkdk*  *[name of the patient; misspelled]*  *I I can see tehstars*  *I can*  *See*  *The*  *Sars*  *You*  *Are y horizon*  *That I I deserve*  *You*  *Are*  *J*  *My*  *Horizon*  *I*  *Used to view you as*  *A*  *A*  *Moutntain*  *There’re areaso many [;aces ive been i\tauatm\9m]\cajt\rekkeber\iits all about presencse*  *Iicqnjustbe hello*  *I*  *Im*  *Justwaking*  *U[*  *I can haer them whisper hahaha* | *Zxcvxc*  *[name of the patient]*  *Kkkkkdddd*  *Laksdjfl;asdkjf*  *I can see the imagesQQQ*  *1*  *Kkjkjkj*  *I see the [microscopy] images I took in 3D*  *Ok*  *Set things aside*  *This is just about me now work through my pain*  *This is good for them to*  *So I’m ok*  *That sip*  *You get them to the rim, then do a sip of a scent and line that up with a uplifting music sound and that’s good*  *Took video*  *I’m going to go into the experience*  *I can do [3D imaging analysis] in my mind*  *The more I engage my consciousness*  *The less I am able to engage the experience*  *The more I engage*  *The more I kinda wake /*  *Nice*  *I see a beautiful sky*  *That means I’m twenty minutes in*  *Interstellar music is good for this*  *I wonder if pre-game*  *Watching beautiful scenery*  *If that influences that experience in a positive way*  *A realization song*  *Could be helpful for patients*  *An “aha” moment*  *To go*  *To give some one*  *Some to give someone that*  *Turning*  *Points*  *In their*  *T*  *Life*  *Get them*  *I*  *A different trajectory*  *“Feels like*  *Feel like just the beat*  *Just the beat inside*  *My soul the*  *Take me to the other side*  *I know*  *Catch me like it’s do or die*  *Take me*  *To the other side”*  *Breaking the fourth*  *Wall*  *At the end of the day I want to*  *Help people*  *And now I’m back to the [microscopy]*  *Explore the universe*  *The music is really good*  *“Catch me like it’s do or die”*  *I can see*  *[name of the provider; misspelled] I want you to be ok*  *I’m gonna go explore*  *Explore*  *Kdkdkdk*  *[name of the patient; misspelled]*  *I I can see the stars*  *I can*  *See*  *The*  *Stars*  *“You*  *Are my horizon*  *That I I deserve*  *You*  *Are*  *J*  *My*  *Horizon*  *I*  *Used to view you as*  *A*  *A*  *Mountain”*  *There’re are so many places I’ve been [uncertain] it’s all about*  *presence*  *I can just be. Hello*  *I*  *I’m*  *Just waking*  *Up*  *I can hear them whisper hahaha* | Patient is fully awake and oriented.  **0 Minutes**  Ketamine infusion initiated (2.0 mg/kg administered IV over 1 hour).  **10 Minutes**  The patient typed his name and hit sequences of keys to orient his hands.  The patient started visualizing images similar to ones he saw while performing microscopy.  The patient chose to focus his attention on improving his mental health  The patient stated that “them” refers to his loved ones, and that by focusing on himself during the experience he was also doing what was best for them.  The patient stated that he was thinking about how to optimize the experience by taking a “sip” of a pleasant scent (i.e. coffee), while listening to music.  The patient asked the provider to take a video for him.  The patient stated that the more he focused on typing, the “less deep” the experience was for him.  **20 Minutes**  The patient placed the song “*Cornfield Chase*” (Zimmer, 2014) from the movie “*Interstellar*” (Nolan, 2014) 20 minutes into his playlist to help him orient.  The patient reflected on possible ways to improve the mental health benefits of ketamine infusions.  The patient transcribed 9 lines from the song “*Every Language is Alive*” by Coldplay, Porter Robinson, Krewella, and Kyante Wilson (Wilson et al., 2019). The tempo was too fast for the patient to transcribe the lyrics with perfect accuracy.  The patient reported that typing to record his experiences felt like “breaking the fourth wall.”  The patient attempted to transcribe one additional line from *Every Language is Alive*” (Wilson et al., 2019).  **30 Minutes**  The patient checked to make sure the provider was doing alright, then decided to focus his attention on his internal experience.  **40 Minutes**  **50 Minutes**  The patient transcribed 4 lines from the song “*Horizon*” by Odsen and Katrine Stenbekk (Odsen & Stenbekk, 2022). The tempo was too fast for the patient to transcribe the lyrics with perfect accuracy.  **60 Minutes**  The ketamine infusion ended.  The patient vocalized a greeting to the provider.  The patient slowly returned to a normal state of consciousness and oriented himself to time and place. |
